# Computer Simulation of Multi-Color Brainbow Staining and Clonal Evolution of B Cells in Germinal Centers

**DOI:** 10.3389/fimmu.2018.02020

**Published:** 2018-09-25

**Authors:** Michael Meyer-Hermann, Sebastian C. Binder, Luka Mesin, Gabriel D. Victora

**Affiliations:** ^1^Department of Systems Immunology, Braunschweig Integrated Centre of Systems Biology, Helmholtz Centre for Infection Research, Braunschweig, Germany; ^2^Institute for Biochemistry, Biotechnology and Bioinformatics, Technische Universität Braunschweig, Braunschweig, Germany; ^3^Centre for Individualised Infection Medicine, Hanover, Germany; ^4^Laboratory of Lymphocyte Dynamics, The Rockefeller University, New York, NY, United States

**Keywords:** germinal center, multiphoton imaging, sequencing, clonal selection, brainbow, computer simulation, mathematical modeling

## Abstract

Clonal evolution of B cells in germinal centers (GCs) is central to affinity maturation of antibodies in response to pathogens. Permanent or tamoxifen-induced multi-color recombination of B cells based on the brainbow allele allows monitoring the degree of color dominance in the course of the GC reaction. Here, we use computer simulations of GC reactions in order to replicate the evolution of color dominance *in silico* and to define rules for the interpretation of these data in terms of clonal dominance. We find that a large diversity of clonal dominance is generated in simulated GCs in agreement with experimental results. In the extremes, a GC can be dominated by a single clone or can harbor many co-existing clones. These properties can be directly derived from the measurement of color dominance when all B cells are stained before the GC onset. Upon tamoxifen-induced staining, the correlation between clonal structure and color dominance depends on the timing and duration of the staining procedure as well as on the total number of stained B cells. B cells can be stained with 4 colors if a single brainbow allele is used, using both alleles leads to 10 different colors. The advantage of staining with 10 instead of 4 colors becomes relevant only when the 10 colors are attributed with rather similar probability. Otherwise, 4 colors exhibit a comparable predictive power. These results can serve as a guideline for future experiments based on multi-color staining of evolving systems.

## 1. Introduction

Permanent multi-color recombination of cells allows monitoring the fate of the stained cells. Cre-dependent recombination of colors based on the Brainbow fluorescent protein reporter construct was applied in the past years to the nervous system (1–4) and to developmental biology (5, 6). As the adopted color is transmitted to the progeny of the cell, this method not only allows to follow the fate of the stained cell itself but also to visualize cell division and the fate of the daughter cells. This particular property of the brainbow allele made it suitable for the study of evolutionary systems like the germinal center (GC) reaction (7), which is an important part of the acute immune response to pathogens (8, 9). While the full repertoire of GC B cells might be assessed in the future at particular time points by sequencing, the brainbow method can be used to monitor the evolution of BC clones in GCs over time (10).

GC reactions are central not only for the clearance of infections but also for generating immune memory. As such they form the basis for the success of vaccinations and are central to the prevention of diseases. The fundamental principle of a GC reaction is an evolutionary process on the scale of a few weeks inside the living organism in lymphoid organs. There, B cells divide and mutate (11) and subsequently undergo a selection process giving rise to high affinity antibodies in response to a pathogenic challenge. The emerging B cells encode a different antibody than their germline counterparts, with better binding properties to the pathogen. The GC reaction is also responsible for a diversification of the pool of antibodies ready to fight against the next infection.

The evolution of B cells in GCs is difficult to monitor. One possibility is sequencing of all B cells at different time points of the reaction (7, 12). Multi-color recombination of B cells bears information of the clonal evolution of the B cells and, thus, would allow us to learn about selection and diversification of B cells. Recent experiments using this approach suggested that B cells are not only optimized for high affinity to the pathogen, as widely accepted, but also for an optimal antibody diversity (7), which was also supported by modeling (13). Here, we analyse the predictive power of the measured color distributions for properties of the GC reaction like clonal dominance and diversification.

## 2. Methods

The *in silico* GC reactions used as the backbone of the present analysis of B cell clonality is fully described in the Supplementary Material. The model architecture is a stochastic event generator with cellular agents in a three-dimensional discretized space. It is complemented by a reaction-diffusion system for chemokines, which are generated and sensed by cellular agents. In addition, each cellular B cell agent carries a position in a shape space, which reflects its similarity to the antibody, which binds optimally to the antigen in question. Somatic hypermutation is modeled as displacement in this shape space. All agents move according to published two-photon measurements. They interact according to the current state-of-the-art model of how GC B cell affinity maturation evolves. Events like movement, division, interaction, and selection are based on rate-derived probabilities per time step, unless stated otherwise (see Supplementary Material). Possible fates of B cells are apoptosis, differentiation to output cells, or recycling to the DZ phenotype (14, 15). The simulations reproduce the population kinetics, affinity maturation, and output cell production in agreement with experimental constraints.

The previously published model (16), was corrected by a substantially higher number of founder cells (7) and was extended by a dynamic-number of division (DND), which states that B cells receiving more signals from T follicular helper cells would divide more (16–18).

### 2.1. Increased number of founder cells

By extrapolation from the number of different founder clones found by sequencing of randomly picked GC B cells to the real number of GC founder clones (7), the old picture of an oligoclonal GC (12, 19) was revised. Instead the number of founder clones was estimated in the range of 100 cells (7). The GC simulation (16) needs to be revised correspondingly. Following Meyer-Hermann and Binder and Meyer-Hermann, A continuous influx of new founder cells during the first days after GC onset is assumed (17, 20). Although influx rates of GC founder cells are currently unknown, in our model we assumed 2 cells per hour limited to the first 4 days of the GC reaction, which generated a number of founder cells consistent with Tas et al. (7). This value might also be estimated with a simple ODE model (see Supplementary Information: *B cell influx rate*).

### 2.2. Color probabilities

The brainbow allele as implemented in the *Rosa26*^Confetti^ allele (1) randomly tags cells with one of 4 different colors. Applying this to both alleles in GC B cells, stains the B cells with one of 10 different color combinations (7). Recombination can be induced prior to the GC reaction or by injection of tamoxifen. In order to simulate the color dynamics in GCs *in silico*, the probability of each color combination was determined as the mean over all GCs in AID-KO experiments, in which mutation and selection are suppressed in GCs (Table 1). The probabilities for 4 color stainings *in silico* were assumed. These values were used in all simulations unless stated otherwise.

**Table 1:** Probabilities of staining B cells with one of either 4 or 10 colors.

**Color**	**4 color**	**Tamoxifen**	**Founder**
Black	0.40	5.210^−1^	1.110^−1^
YFP	0.18	1.310^−1^	1.710^−1^
RFP	0.18	1.210^−1^	1.710^−1^
CFP	0.18	1.010^−1^	1.710^−1^
GFP	0.06	5.610^−2^	5.110^−2^
C/R	–	2.710^−2^	8.010^−2^
C/Y	–	1.610^−2^	8.010^−2^
Y/R	–	1.410^−2^	2.810^−2^
G/R	–	3.210^−3^	8.010^−2^
C/G	–	1.810^−3^	2.810^−2^
G/Y	–	1.010^−3^	2.810^−2^

*For 10 colors, the probabilities in tamoxifen-induced and founder cell staining experiments were distinguished and derived from AID-KO experiments [(7), renormalized to an amount of black of 52%]. Black denotes the probability of no staining. Values rounded to two digits*.

### 2.3. Delayed action of tamoxifen

Injection of tamoxifen induces Cre-lox recombination of one or two alleles. The GC B cell then expresses one of ten possible color combinations. Tamoxifen-activity continues for a finite time. An exponential decay of tamoxifen and, consequently, of the recombination probability was assumed *in silico*. The initial probability *p*_stain, 0_ of tamoxifen-induced recombination is not known. It was chosen such that the experimentally observed fraction of stained cells *f*_stained_, which is known from experiment (see Table 1, one minus black).

The initial probability of recombination *p*_stain, 0_ after injection of tamoxifen is estimated with the help of a simplifying model. Staining is initiated in the simulation in time steps Δ*t*_stain_. With the tamoxifen decay time τ_tamoxifen_, the fraction of stained cells can be approximated as:

(1) $$\begin{array}{l} f_{\text{stained}}=\frac{p_{\text{stain},0}}{\Delta t_{\text{stain}}}\int_{0}^{\infty }\exp\left(-\frac{t}{\tau_{\text{tamoxifen}}}\right)dt\\ =\frac{p_{\text{stain},0}}{\Delta t_{\text{stain}}}\tau_{\text{tamoxifen}}. \end{array}$$

Note that this holds only for probabilities *p*_stain, 0_, sufficiently small such that double staining can be neglected.

For practical reasons, tamoxifen activity was stopped at time τ_stainstop_. This modifies Equation (1) to

(2) $$\begin{array}{l} f_{\text{stained}}=\frac{p_{\text{stain},0}}{\Delta t_{\text{stain}}}\int_{0}^{\tau_{\text{stainstop}}}\exp\left(-\frac{t}{\tau_{\text{tamoxifen}}}\right)dt\\ =\frac{p_{\text{stain},0}}{\Delta t_{\text{stain}}}\tau_{\text{tamoxifen}}\left(1-\exp\left(-\frac{\tau_{\text{stainstop}}}{\tau_{\text{tamoxifen}}}\right)\right). \end{array}$$

This condition approximates the initial staining probability *p*_stain, 0_ to

(3) $$\begin{array}{ll} \frac{p_{\text{stain},0}}{\Delta t_{\text{stain}}} & =\frac{f_{\text{stained}}}{\tau_{\text{tamoxifen}}\left(1-\exp\left(-\frac{\tau_{\text{stainstop}}}{\tau_{\text{tamoxifen}}}\right)\right)}. \end{array}$$

This relation was used in the simulations in order to fix *p*_stain, 0_ with τ_tamoxifen_ = 24 h, τ_stainstop_ = 2 days, and *f*_stained_ equal to 1 minus black in Table 1. In order to save computation time in the simulations, the staining procedure is called with Δ*t*_stain_ = 1 h. In the simulations, a color is only attributed once to a cell, unless stated otherwise, i.e., in the case of an attempt to restain an already stained cell, this attempt is ignored.

## 3. Results

### 3.1. Correlation of clonal and color dominance

It is known that high affinity B cells emerge from the GC reaction in a process of cycles of mutation and selection. The dynamics of clonal selection and shift toward high affinity clones in the course of the reaction was recently analyzed with the help of random attribution of colors to either GC founder cells or to GC B cells in an early phase of the reaction (7). This allowed them to follow GC B cells of a particular color and was interpreted to provide information on the clonal evolution of GC B cells. In particular, the largest fraction of cells stained by a single color, short the *color dominance*, was considered as a measure of the *clonal dominance*, i.e., the largest fraction of GC cells that stem from a single clone (see Table 2). A clonal dominance of 100% would correspond to all GC B cells being derived from a single clone, which would be the result of strong selection of an advantageous clone. The smaller the clonal dominance, the more different B cell clones coexist in the same GC.

**Table 2:** Definition of the terminology used throughout.

**Term**	**Symbol**	**Definition**
Staining		Cre-lox recombination of the brainbow alleles (one or both) inducing a constitutive expression of a color in the cell that is transmitted to its progeny. In the simulation this corresponds to the simple attribution of a color to a cell object. The probabilities to induce particular colors are listed in Table 1.
Colors	*C*	The set of 4 or 10 colors (excluding black) that can be attributed to cells.
Clones	*F*	All GC founder cell define the set of GC clones.
Lineages	*L*_*t*_0__	All GC B cells present in the GC at a particular time point *t*_0_ (typically the time point of giving tamoxifen) during the GC reaction define the set of GC B cell lineages *L*_*t*_0__ with *N*(*t*_0_) elements. Note that two daughter cells of a clone may give rise to different lineages.
Cell numbers	*N*(*t*)	The total number of GC B cells at time *t* of the GC reaction.
	*F*_*i*_(*t*)	The number of GC B cells at time *t* that stem from clone *i*∈*F*.
	*L*_*i*_(*t*)	The number of GC B cells at time *t*>*t*_0_ that stem from a lineage *i*∈*L*_*t*_0__.
	*C*_*i*_(*t*)	The number of GC B cells at time *t* that express a color *i*∈*C*.
Clonal dominance	$F\left(t\right)=\underset{i\in F}{\max}\left\{\frac{F_{i}\left(t\right)}{N\left(t\right)}\right\}$	The largest fraction of the GC B cells at particular time points *t* during the GC reaction that stems from a single clone among all clones.
Lineage dominance	$L\left(t\right)=\underset{i\in L_{t_{0}}}{\max}\left\{\frac{L_{i}\left(t\right)}{N\left(t\right)}\right\}$	The largest fraction of the GC B cells at particular time points *t*>*t*_0_ that stems from a single lineage among all lineages.
Color dominace	$C\left(t\right)=\underset{i\in C}{\max}\left\{\frac{C_{i}\left(t\right)}{N\left(t\right)}\right\}$	The largest fraction of the GC B cells that express a single non-black color.
Color density	$D\left(t\right)=\underset{i\in C}{\sum }C_{i}\left(t\right)$	The fraction of GC B cells that express any (non-black) color.
Staining threshold	*T*	The staining threshold *T* restricts the analysis to only those GCs with the property that the color density at the time *t* of analysis is above the staining threshold, i.e., *D*(*t*)>*T*.
PDD	*C(t)* *D*(*t*)	The product of color dominance and color density.

Here, we replicate these experiments *in silico*, and determine under which conditions the evolution of clonal and color dominance in GCs is correlated and delineate the limits of this correspondance as a guideline for future experiments. The analysis was restricted to the dominant color because, in our hands, the inclusion of the second most dominant color did not improve the results.

### 3.2. Staining of founder cells

**Figure 1:**
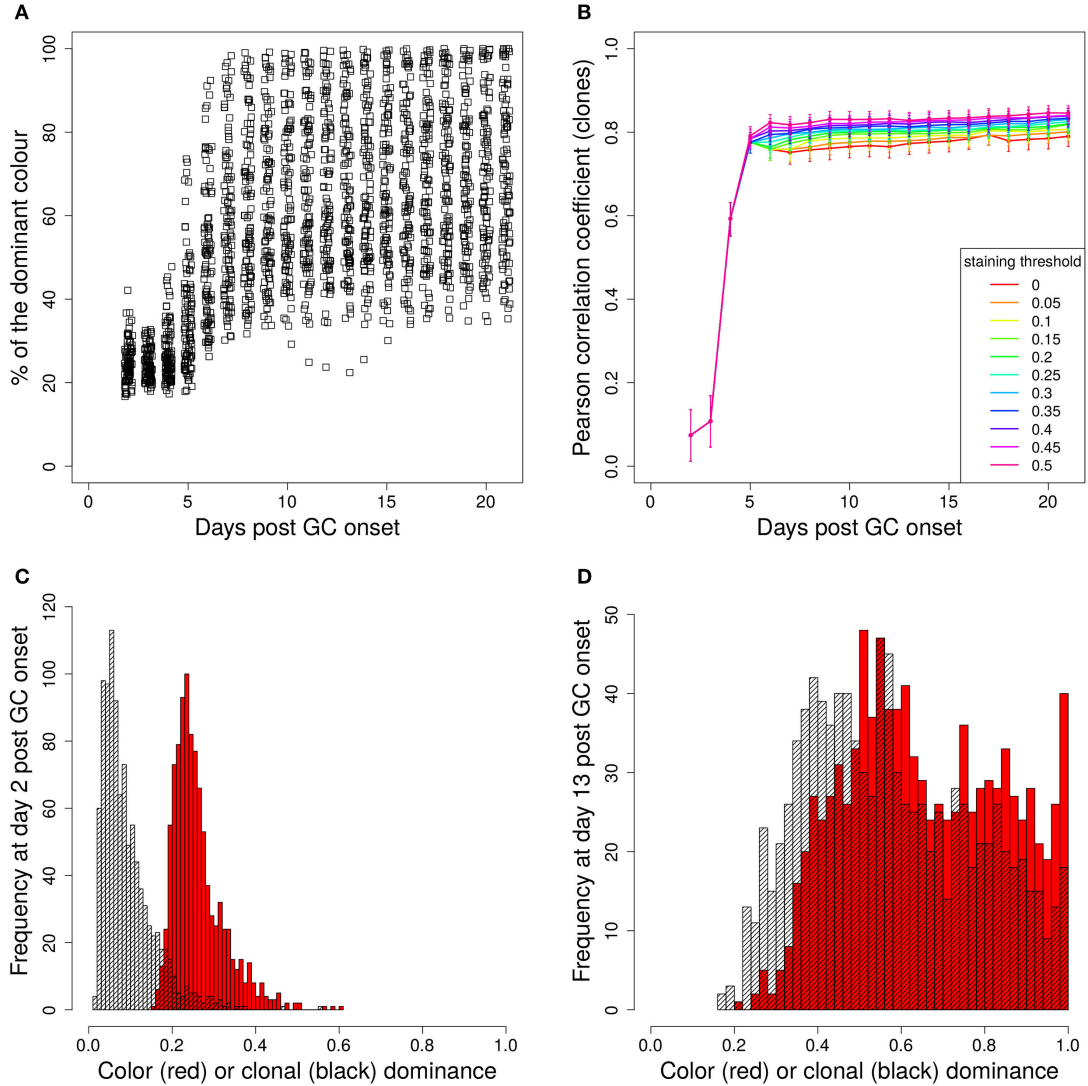
Staining of GC founder cells guarantees a good correlation of color and clonal dominance. The GC founder cells were stained with 10 colors before the GC reaction. The size of the largest color **(A)** (first 100 out of 1,000 GC simulations) and its correlation with the clonal dominance **(B)** were monitored over the duration of the GC reactions. The impact of imposing a staining threshold onto the correlation was tested in **(B)** (colors). Pearson correlation coefficient from 1,000 *in silico* GCs. 95% approximate confidence intervals to the Pearson product moment correlation were computed using the Fisher transformation. The distribution of color (red) and clonal (black) dominance are shown at day 2 **(C)** and 13 **(D)** post GC onset.

In a setting in which B cells are stained before the GC reaction, most B cells entering the GC already carry a color (Table 1 column *founder*). This corresponds to spontaneous recombination of B cells in the Mx1-Cre-mice in Tas et al. (7) and allows monitoring the clonal evolution inside GC reactions based on the evolution of color dominance. However, this relies on a good correlation between clonal and color dominance. We replicated the color dynamics *in silico* (Figure 1A). The color dominance starts from a baseline level, which is basically reflecting the probability distribution of getting the different colors (Table 1). Around day 5 post GC onset, GCs differ markedly in the fraction of cells expressing the dominant color, which is at the time after B cell expansion when the selection pressure on BCs is getting strong. The diversity of color dominance reaches a saturated level around day 8 post GC onset, which coincides with the time associated of take over of high affinity clones (21), and is kept by the end of the GC reaction.

In the simulations, full information on the clonal evolution is known as well, which puts us in the position to determine the degree of correlation between the color and the clonal dominance (Figure 1B). The correlation is sufficiently strong to allow for an association of clonal with color dominance. It was tested whether imposing a threshold staining level for each GC to be included in the analysis would change the correlation. The level of correlation was rather independent of this threshold (Figure 1B).

This correlation can be confirmed by the explicit comparison of the color and the clonal dominance (Figures 1C,D). During expansion at day 2 post GC onset, the B cells only divided and mutated but only underwent selection processes in rare cases. As a consequence, clonal dominance is rather low and color dominance reflects the staining probabilities. Both peaks are clearly seperated. Later at day 13 post GC onset when affinity maturation is accomplished, both distributions largely overlap. However, it can also be seen that the color dominance has the tendency to over-estimate the clonal dominance.

### 3.3. Tamoxifen-induced staining of cells

Next, we investigated the attribution of colors to B cells at day 2 post GC onset, which corresponds to tamoxifen induced recombinations in AID-CreERT2 mice (7). At day 2 of the reaction, founder clones already expanded and diversified their encoded B cell receptor by somatic hypermutations. This leads to a random staining of many copies of cells descendent of the same initial founder clone. Thus, a correlation of cell color with B cell clones is not expected.

**Figure 2:**
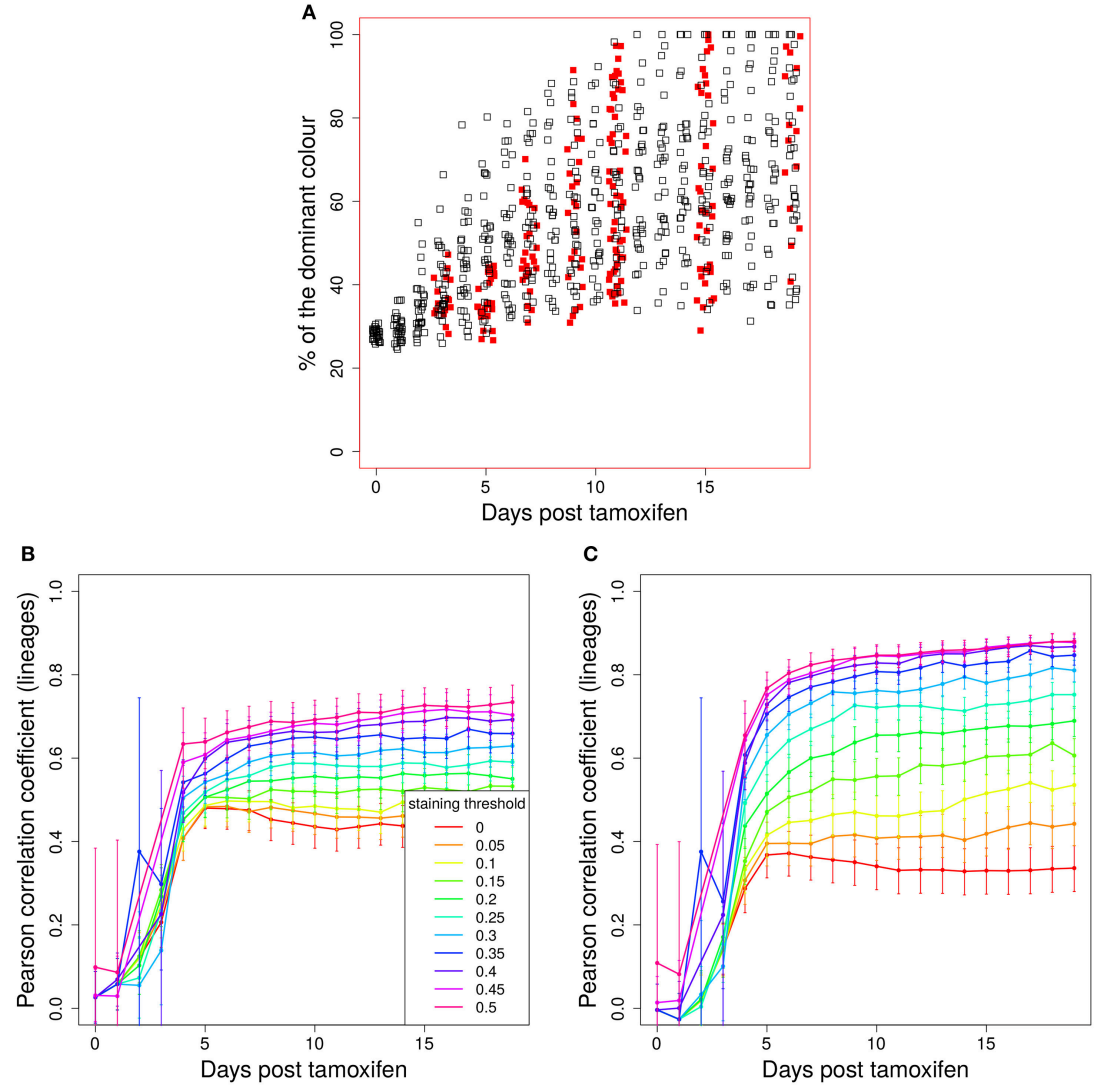
Predictivity of tamoxifen-induced staining. **(A)** Evolution of color dominance in response to tamoxifen-induced staining. Thirty simulations (black open squares) are compared to the experimental color dominance behind Figure 3F in Tas et al. (7) (closed red squares). Correlation between lineage dominance and **(B)** color dominance or **(C)** the product of color dominance and color density (PDD). Different staining thresholds were distinguished (line colors). Following tamoxifen induced staining as in Table 1, GC B cells were stained at day 2 post GC onset with 10 colors. Pearson correlation coefficient from 1,000 *in silico* GCs. 95% approximate confidence intervals to the Pearson product moment correlation were computed using the Fisher transformation.

The evolution of the color dominance *in silico* and *in vivo* is compared in Figure 2A. As in Figure 1A, the color dominance starts from a baseline and increases over time. While the overall agreement between theory and experiment is convincing, there is a small subset of GC simulations with a higher color dominance at days 5 and 7 post tamoxifen. This might be a hint to an overestimation of the selection pressure or of the GC diversity *in silico*. Note, that the number of *in silico* GCs is higher than *in vivo* at both days.

B cells stained in the course of a reaction each define a new lineage. The evolution of colors is now interpreted to provide information on the evolution of those lineages. Indeed, a correlation between color and lineage dominance exists (Figure 2B, red line). However, it is much weaker than in the case of founder cell staining and limits the interpretation of the data on the evolution of color dominance.

#### 3.3.1. A staining threshold guarantees a correlation of color and lineage dominance

We sought for a possible filter for the *in silico* GC data that improves the correlation between lineage and color dominance. Given that a large proportion, in the range of 50% of lineages, is not stained by tamoxifen *in silico* and *in vivo*, there is a substantial fraction of GCs that are dominated by a black lineage. This results in an underestimation of the lineage dominance by the color dominance. Indeed, the introduction of a staining threshold, i.e., a minimum fraction of total stained cells in each *in silico* GC that has to be reached for inclusion of the GC in the analysis, substantially increases the correlation between lineage and color dominance (Figure 2B, blue and magenta lines). However, the level of correlation was still not comparable to that observed when staining founder cells before entering the GC reaction.

The lineage dominance is also (weakly) correlated to the fraction of stained cells in a GC (Figure S2), referred to as color density in the following (see Table 2), provided a staining threshold is imposed. This is the case because a staining fraction above the initial mean staining level is more likely to occur for GCs with high lineage dominance. We tested the degree of correlation between lineage dominance and the product of color dominance and color density, shortly denoted as *PDD* in the following (Figure 2C). PDD approximates the normalized density score (NDS), which was used *in vivo* and is defined as the product of color dominance with the density of colored cells in the dark zone, measured as number of colored cells per 10μm^2^ (7). While the correlation is even weaker in GCs with low color density (yellow, orange, and red lines), the staining threshold allows to reach the same high degree of correlation as was found for the staining of founder cells. Thus, we recommend to use a staining threshold in the range of 40%. With lower thresholds, the correlation gets weaker. With higher thresholds, the correlation hardly improves. Instead, the statistics get critical because a large fraction of GCs is left out of the analysis.

**Figure 3:**
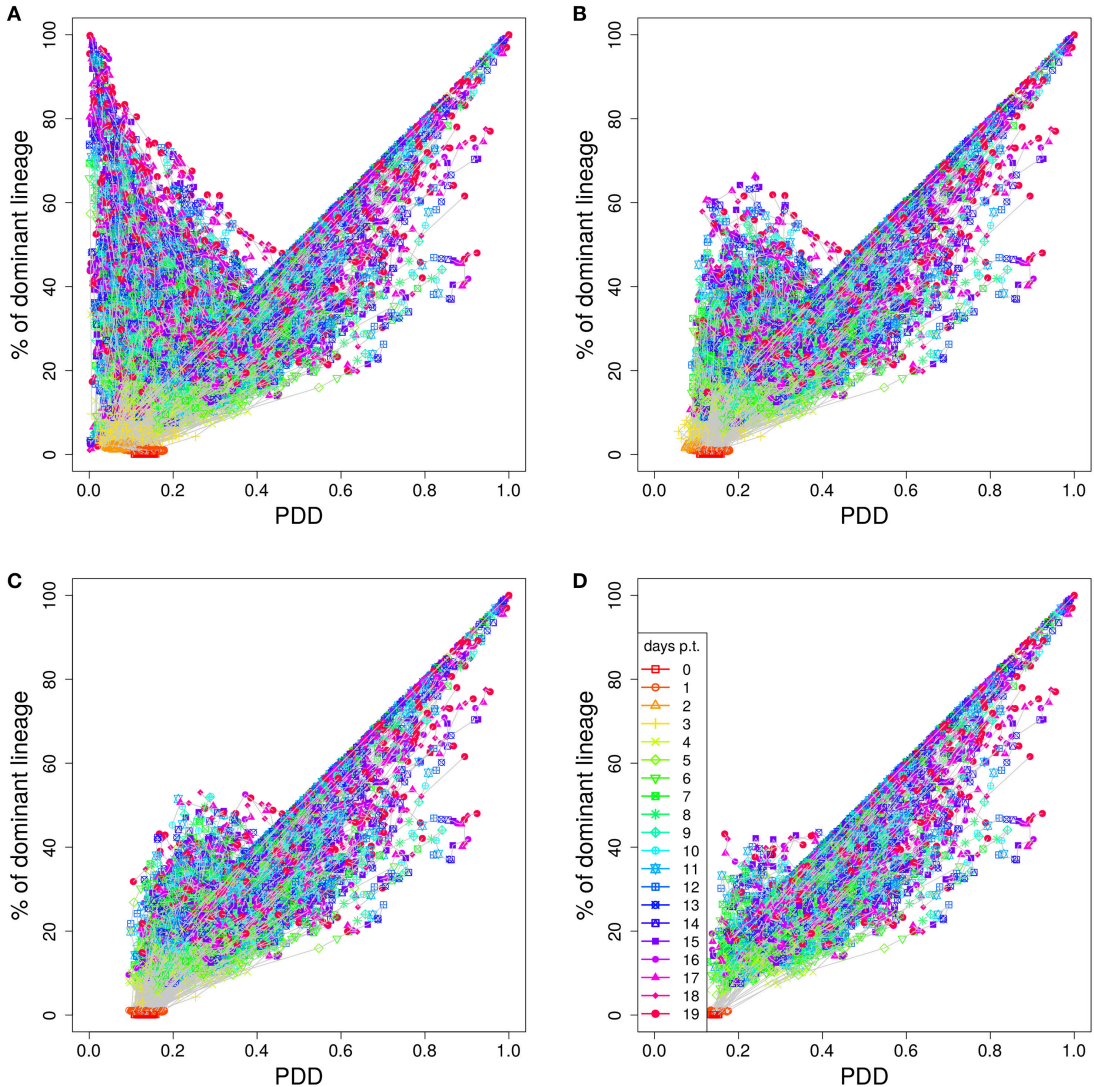
Higher staining thresholds eliminate GCs dominated by unstained lineages. Lineage dominance vs. the product of color dominance and staining density (PDD) with a staining threshold of 0 **(A)**, 30 **(B)**, 40 **(C)**, and 50% **(D)**. GC days are distinguished by symbol/line colors. GC B cells were stained at day 2 post GC onset with 10 colors (Table 1) in 1,000 *in silico* GCs.

In order to illustrate how the removal of GCs dominated by black, i.e., not stained, B cells improves the correlation we plot lineage dominance against color density (Figure 3). The perfect correlation would correspond to all symbols being concentrated on the diagonal line. The symbols above the diagonal are those with a low color dominance but high lineage dominance. These can be attributed to the cases described above when the staining procedure failed to stain the lineage which became dominant later on during the GC reaction. These GCs are dominantly black. The introduction of a threshold removes these GCs from the analysis as can be seen by the reduction of the number of GCs above the diagonal line with increasing threshold. Note that a staining threshold of 50% also eliminates a part of the GCs at the day of staining (day 0, red symbols) from the analysis, which by definition exhibit a staining level of 48%. By random fluctuations, there is still a subset of GCs with a staining level above the threshold of 50%. The impact of increasing the threshold from 40 to 50% on later time points of the GC reaction is comparably weak, which explains the minor change in the correlation in Figure 2C.

The graph also illustrates that the product of color dominance and color density has the tendency to overestimate lineage dominance. These are the GCs with symbols below the diagonal line in Figure 3. Overestimation is a result of different persisting lineages being stained with the same color. If this happens, different lineages contribute to the same color, while the lineage dominance only corresponds to one of those lineages. This effect is less pronounced for large PDD, as was confirmed by sequencing *in vivo* (7). The introduction of a staining threshold to select a subset of GCs for analysis makes the fraction of GCs with an overestimation of the lineage dominance more prominent.

#### 3.3.2. The dominant color switches during GC selection

**Figure 4:**
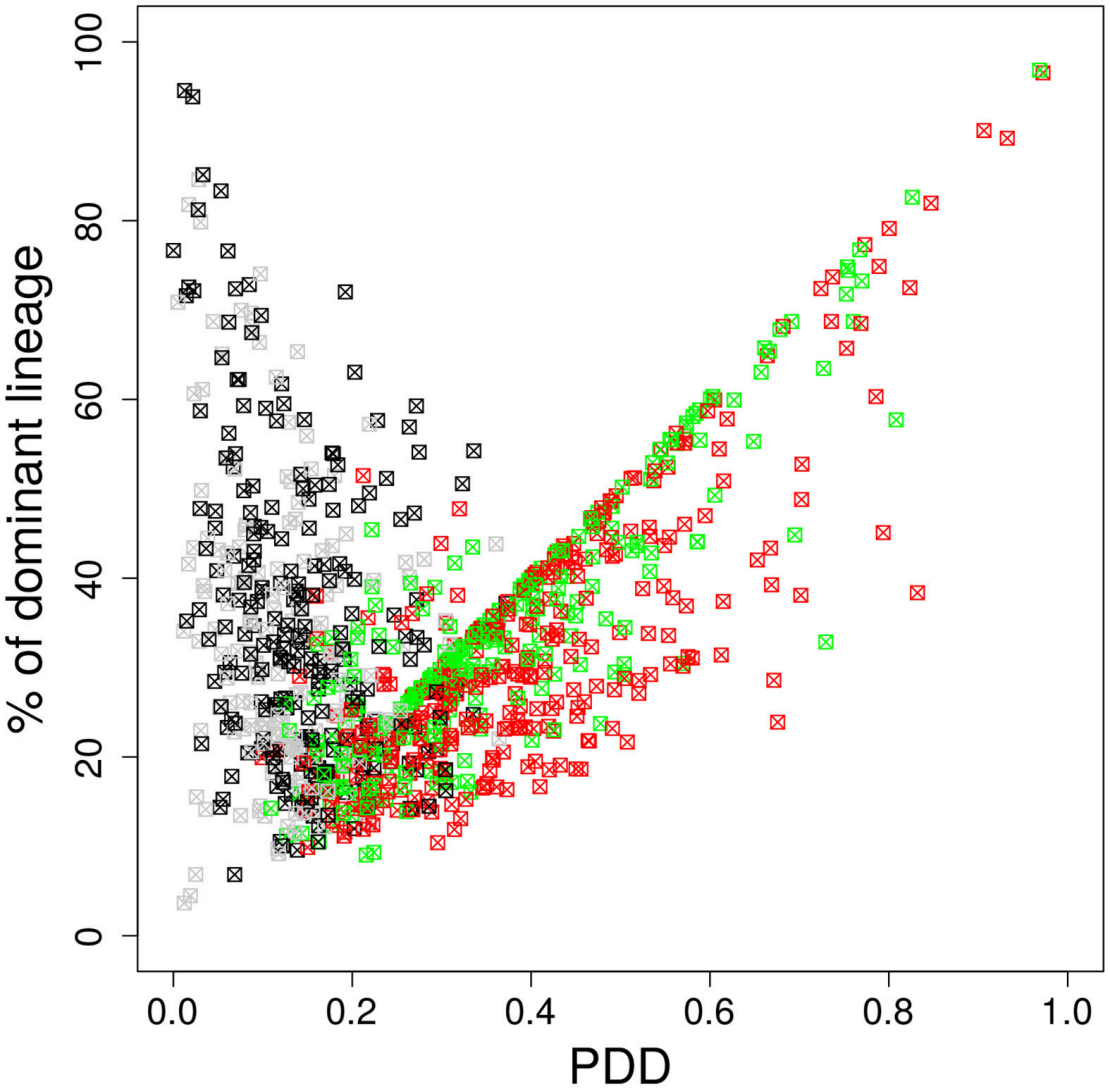
The dominant color switches in the course of GC affinity maturation. Lineage dominance vs. the product of color dominance and staining density (PDD) with a staining threshold of 40% at day 11 post staining (subset of data in Figure 3). Black and gray squares are GC reactions with a staining level below the threshold, while red and green ones are above the threshold, thus, kept for analysis. Black and red GCs have the property that the initially dominant color was also dominant at day 11 post staining. Thus, in gray and green GCs the dominant color has switched between the time of staining and the time of analysis. GC B cells were stained at day 2 post GC onset with 10 colors (Table 1) in 1,000 *in silico* GCs.

It is possible that the inhomogeneous color distribution to the B cells (Table 1) is dominating the subsequent fate of the color dominance. If the color reflects the progression of the selection process during the GC reaction, the dominant color should switch between the time point of staining and the time point of evaluation when the selection of lineages was completed. We assumed that this would be the case at day 11 post staining (21). Indeed, a large fraction of GCs switched the dominant color between the time of staining and day 11 (Figure 4). This result further supports that the analysis of color distributions is a suitable measure for the analysis of selection in GCs.

#### 3.3.3. The time of tamoxifen-induced staining is important

**Figure 5:**
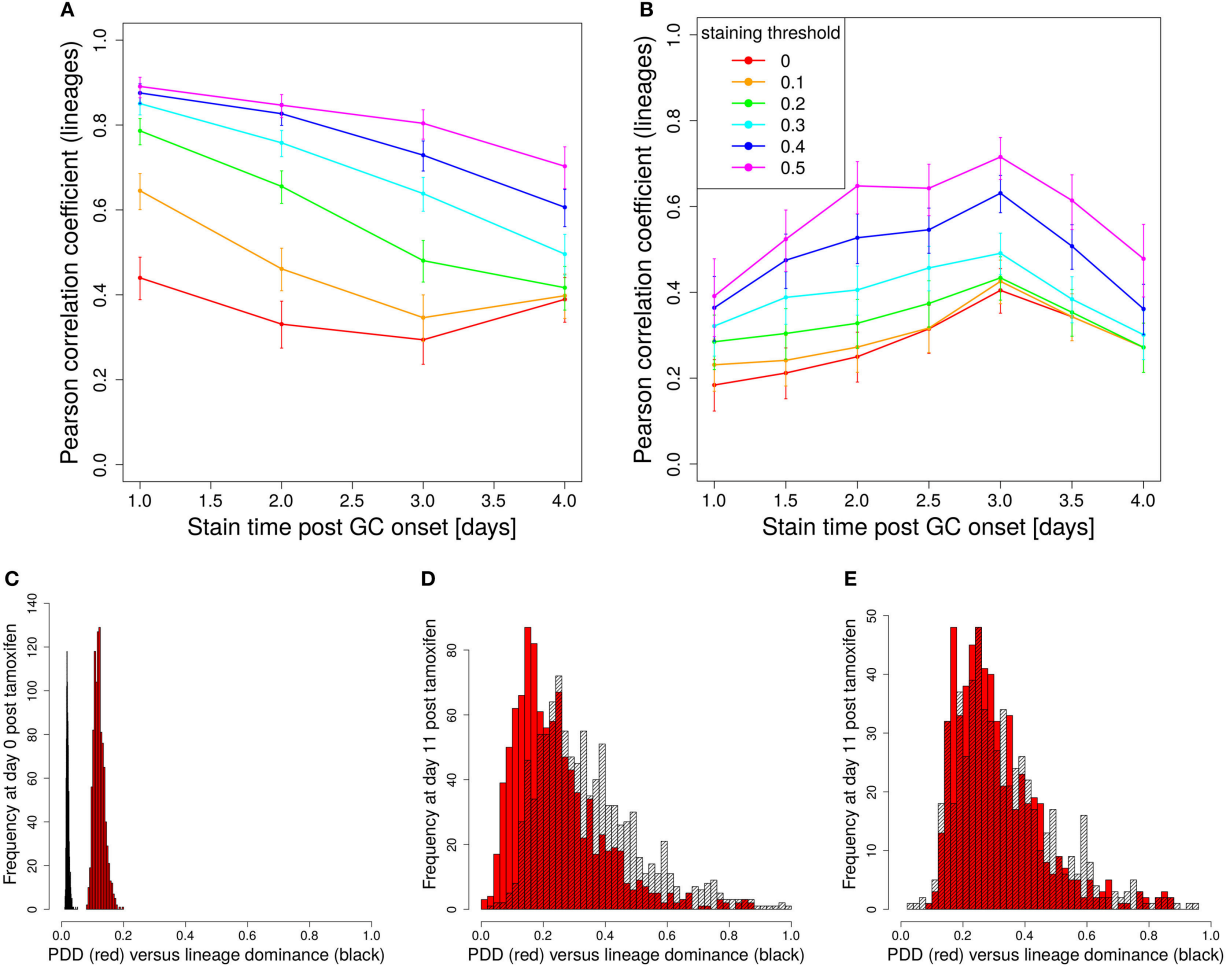
The staining time point determines the predictive power. Correlation between lineage and the product of color dominance and color density in dependence on the time point of staining. Simulations with single shot **(A)** or dynamic decay **(B)** of tamoxifen-induced staining with 10 colors (Table 1). For dynamic tamoxifen decay in **(B)**, a half life of τ_tamoxifen_ = 24 h was assumed in Equation (3). Lineages were defined at the time of tamoxifen injection. For lineages complemented by all founder cells entering the GC after injection see Figure S1. For dynamic tamoxifen decay with tamoxifen given at day 2 post GC onset, the lineage dominance distribution is compared to the distribution of the product of color dominance and color density (PDD) without threshold at day 2 **(C)** and 13 **(D)** and with a staining threshold of 40% at day 13 post GC onset **(E)**. Data in **(A,B)** show the Pearson correlation coefficient from 1,000 *in silico* GCs at day 11 post tamoxifen with different staining thresholds (line colors). 95% approximate confidence intervals to the Pearson product moment correlation were computed using the Fisher transformation.

The time of lineage definition by tamoxifen is critical for the analysis. For one shot stainings it holds that the earlier recombination is induced the better the correlation with the lineage dominance (Figure 5A), provided we use a staining threshold of 20% or higher. Two lineages that will survive on long-term might be stained with the same color, which overestimates the lineage dominance. At day 3 or 4 of the GC reaction, many low affinity B cells were already eliminated such that the fraction of long-term survivor lineages increases at the time of staining. Hence, an attribution of the same color to different long-term surviving lineages becomes more probable the later the staining is induced.

In addition, lineages become less dominant the later they are defined. This is because similar variants of potentially dominant lineages are defined as different lineages, although neither has a fitness advantage over the other. Hence, it is unlikely that one of them gets lost during further selection. The later staining is induced, the more probable it is to define two similar lineages as different lineages. As a consequence, lineage dominance gets more and more limited the later staining is induced (Figure S3). While with a lineage definition at day 1 post GC onset 100% lineage dominance is frequent, at day 4 the largest dominance found in 1,000 GC simulations was at 60%.

### 3.4. Decay of tamoxifen-induced staining of cells

The staining induced by tamoxifen is not a one shot event. Upon tamoxifen injection, recombination of B cells can be induced for a limited time and the probability of recombination decreases over time. The detailed dynamics of the reduction of the recombination probability is not known. We assumed an exponential decay (Equation 3), which reflects a linear decay of tamoxifen activity. As we are interested in the correlation of the color dominance with the dominance of the lineages existing at the time of tamoxifen injection, we decided to define a new lineage not at the time of color attribution but at the time of tamoxifen injection. The correlation between lineage dominance and the product of color dominance and color density is reduced in absolute terms when such staining dynamics are included (Figures 5A,B). The comparison of the distribution of lineage dominance and PDD shows how the two distributions approach each other in the course of GC development but stay separate with a substantial fraction of GCs under-estimating the lineage dominance (Figures 5C,D). This can be repaired by imposing a staining threshold of 40% (Figures 5E).

#### 3.4.1. The set of lineages depends on the time of tamoxifen injection

The marking difference to the one-shot staining is the existence of a time point between day 2 and 3 post GC onset at which correlation is maximized. A population of GCs not observed in one-shot stainings and characterized by low lineage dominance and high color dominance emerges. This new GC population exists for early stainings only (day 1 or 2 post GC onset) and is robust against staining thresholds (Figure S4). It is associated with founder cells entering the GC reaction after the starting time of staining. Indeed, if these late founder cells are included in the set of lineages, this population of GCs disappears again together with the optimal time of staining (Figure S1). At late staining times, correlation is lost due to staining ambiguities. At early staining times, it is lost because of stained cells not belonging to any monitored lineage, giving rise to the observed optimal time point for initiation of B cell lineage staining (Figure 5B). This result emphasizes that it is important to consciously chose the time point of tamoxifen injection because it impacts on the resulting set of lineages and may change the interpretation of the experimental results.

#### 3.4.2. A decay of the staining probability effectively retards staining

As described for the one-shot staining scenario, there is a general tendency that later staining reduces the correlation. By the decay of tamoxifen-induced staining activity, staining is distributed onto 2 days, which corresponds effectively to a retardation of staining by roughly 1 day. Indeed, inititation at day 3 with tamoxifen decay is effectively rather similar to initiation at day 4 with one-shot staining (Figure 5). This effective retardation of staining reduces the overall level of correlation.

#### 3.4.3. The impact of recombination of already recombined cells

During the time period of tamoxifen activity, it is possible that a cell undergoes multiple recombination events. This would imply that a cell already expressing a color may switch color. Here, we investigated whether this process would impact on the interpretation of color dominance in terms of lineage dominance. When restaining of already stained cells was allowed *in silico*, the resulting correlation between lineage dominance and color dominance is further reduced (Figures S5A,B). However, only a small impact was found on the correlation between lineage dominance and PDD (Figures S5C,D). While the possibility of ongoing and repetitive recombination makes the resulting color dominance more fuzzy, the correlation with PDD appears robust.

#### 3.4.4. A decay of the staining probability induces a fuzzy color distribution

The resulting distribution of colors at the end of the staining procedure is less well defined compared to the one-shot staining, where the distribution reflects the color probabilities (see Table 1). With tamoxifen decay, a lineage stained right at the beginning of the extended staining period will also stain all of the progeny of this lineage. In contrast, for a lineage stained at the end of the staining period, only a small subset of the progeny is stained, for the cell defining the lineage has divided a number of times and only one of these daughter cells is stained together with its progeny. Other progeny from the very same lineage might not be stained or be stained with different colors. As a consequence, the color dominance under-estimates the lineage dominance (Figure S3). Thus, at the end of the staining period, the number of stained cells from a lineage does not necessarily reflect the size of the lineage and adds to the uncertainties associated with staining different lineages with the same color (over-estimation) or staining the same lineage with different colors (under-estimation). This limitation gets even more important for late initiation of staining, when it gets more likely that two similar parts of a lineages both survive GC selection. The higher variability of the color distributions at the end of the staining process, overall reduces the correlation of color and lineage dominance.

#### 3.4.5. A shortened tamoxifen staining activity would improve color analysis

Some of these stained GCs under-estimating lineage dominance are removed by the staining threshold (Figures 5D,E). As tamoxifen activity was assumed to decay exponentially, in many cases staining happens only one division after the time of initiation of staining, which may still induce a high degree of staining. Also the staining of other progeny from the same lineage with a different color would keep the stained cell fraction high. Thus, a relevant proportion of GCs exists, which exhibits a high fraction of stained cells above the staining threshold but still results in an under-estimate of lineage dominance (Figure S4). A slow decay of tamoxifen activity in the range of days, requires a higher staining threshold for the analysis of 50% or higher in order to keep up with the correlation level of one-shot stainings. In view of the reduced statistics (see absolute counts of GCs in Figures 5D,E), it would be more advantageous to stop tamoxifen activity at defined time points in experimental settings. For example, one might consider shortening the period of tamoxifen activity by administering the drug already in its active form as 4-hydroxy-tamoxifen.

#### 3.4.6. Later staining limits lineage dominance

The later the lineages are defined, the more the lineage dominance achieved is limited (Figure S3). While lineages fully dominating the GCs exist for stainings initiated at day 1–3 post GC onset, their frequency decreases. In stainings at day 4, the highest dominance is reduced to 60%. For late stainings, the identification of GCs dominated by single clones becomes rare based on tamoxifen-induced staining.

### 3.5. Colored GCs are predictive of black GCs

Imposing a staining threshold in the range of 40% or higher turned out to be critical in order to ensure predictive power of the color dominance for the lineage dominance. This was tested for the GCs satisfying the staining threshold. It would be of particular interest, whether the statements on lineage dominance are not only valid for the highly stained GCs but apply to all GCs.

**Figure 6:**
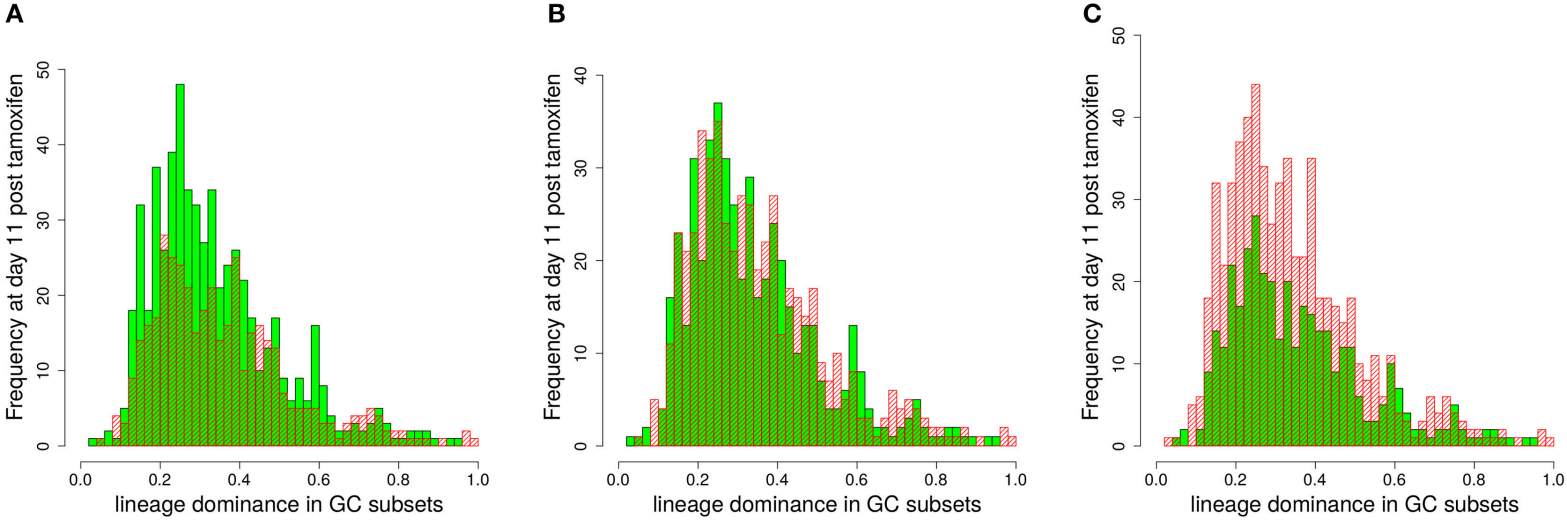
Estimation of the lineage dominance of GCs below the staining threshold. The distribution of lineage dominance is shown for the GCs above (green) and below (red) the staining threshold separately. Data were derived from the 1,000 simulations with dynamic tamoxifen decay as described in Figure 5 at day 11 post tamoxifen. The comparison of the GC subsets was repeated for staining thresholds of 40 **(A)**, 45 **(B)**, and 50% **(C)**.

In order to test this, we compared the lineage dominance distribution for GCs above and below the staining threshold (Figure 6). There exists an optimal staining threshold of 45% for which the lineage dominance of the GCs kept (green bars) and deleted (red bars) from the analysis are widely identical (Figure 6B). With higher staining thresholds (Figure 6C), lineage dominance in the range of 30% are more frequent in the GC subset deleted from the analysis. Thus, the GC subset kept for analysis will underestimate the lineage dominance in this regime. For lower staining thresholds (Figure 6A), the situation is inverted. The difference between both subsets scales with the deviation of the staining threshold from 45%. The optimal staining threshold of 45%, shown in Figure 6 for the case of decaying tamoxifen activity, equally holds true for one-shot stainings (data not shown). In conclusion, the staining threshold of 45% not only guarantees a fairly good correlation with lineage dominance with acceptable statistics. It also guarantees that the lineage dominance estimated with the subset of GCs stained to more than 45% remains valid for the whole set of GCs.

### 3.6. Impact of the fraction of stained cells

**Figure 7:**
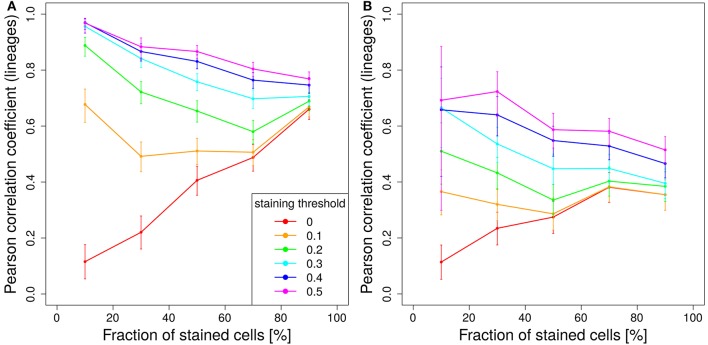
Staining less cells improves predictive power. Correlation between lineage and the product of color dominance and color density in dependence on the fraction of stained cells. Simulations with single shot staining **(A)** or dynamic decay **(B)** with 10 colors (Table 1) at day 2 post GC onset. Pearson correlation coefficient from 1,000 *in silico* GCs at day 11 post tamoxifen with different staining thresholds (line colors). 95% approximate confidence intervals to the Pearson product moment correlation were computed using the Fisher transformation.

The fraction of stained GC B cells induced by tamoxifen was in the range of 50% *in vivo* (7). It is not clear whether a different fraction of stained cells would improve the correlation between color and lineage dominance or reduce it. An increase of the stained fraction would allow to achieve better statistics. We varied the fraction of stained cells between 10 and 90% (Figure 7). Without imposing a staining threshold the intuitive result that staining more infers a better correlation of color and lineage dominance is confirmed (Figure 7, red lines).

This relationship is turned around for higher staining thresholds. The lower the stained fraction of GC B cells the better the color dominance informs about the lineage dominance. Compared to 50% stained cells with a staining threshold of 40%, the same correlation is found for 10% stained cells with a staining threshold of less than 20% (Figure 7A). There is a trade-off between less stained cells, which reduces the statistics, and a lower cut-off, which increases the statistics again. Experiments should be planned to stain a comparably low fraction of cells in order to facilitate the analysis and to improve the predictive power of the color distribution. Staining of lower fractions of cells requires less repetitive dosing of tamoxifen, such that the limitations due to extended staining periods (Figure 5) are also reduced.

The same trend holds true for a tamoxifen activity spread over 2 days. However, the overall level of correlation is lower (Figure 7B).

Tamoxifen-induced cell staining induces a color distribution that carries information on the lineage distribution but not on the clones. Information on the founder cells is widely lost. In the limit of low staining fractions with high staining thresholds it is possible to get a fair correlation of early one-shot induced color dominance and clonal dominance (Figure S6). However, already at day 2 the correlation becomes rather weak.

### 3.7. The predictive power of 4 and 10 colors is similar

**Figure 8:**
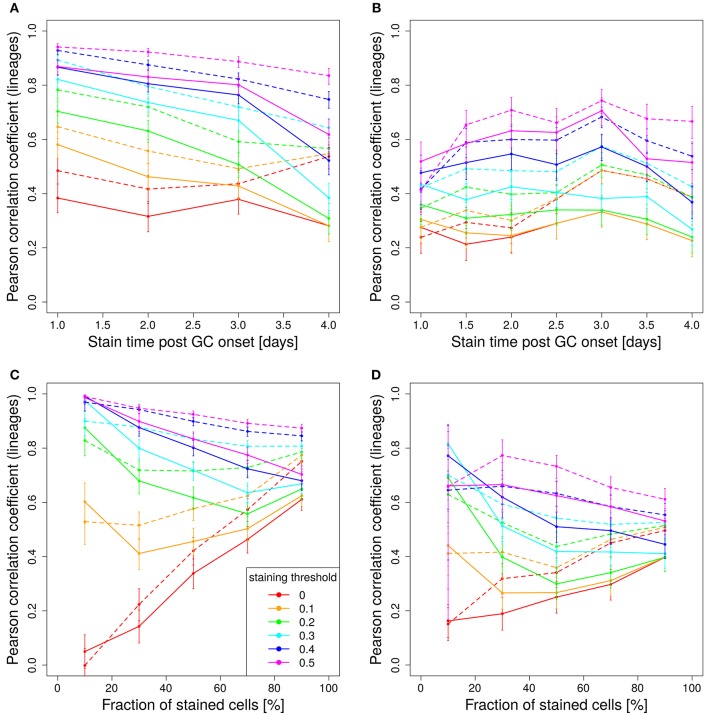
Four colors have similar predictive power than 10 colors. Correlation between lineage and the product of color dominance and density in dependence on the time point of staining **(A,B)** or the fraction of stained cells **(C,D)**. Simulations with single shot **(A,C)** or dynamic decay **(B,D)** of tamoxifen-induced staining with 4 colors (Table 1) (full lines) or with 10 colors with equal probabilities (dashed lines). For dynamic tamoxifen decay, a half life of τ_tamoxifen_ = 24 h was assumed in Equation (3). Lineages were defined by the cells present at the time of tamoxifen injection. Pearson correlation coefficient from 1,000 *in silico* GCs at day 11 post tamoxifen with different staining thresholds (line colors). 95% approximate confidence intervals to the Pearson product moment correlation were computed using the Fisher transformation.

In experimental settings, the variety of colors is generated making use of the so-called brainbow allele (1), which was implemented in the Rosa26^Confetti^ mice (5). The random Cre-recombination of the color segments is induced by injection of tamoxifen and generates cells with 4 different colors. When the brainbow staining is implemented on both alleles, the diversity of colors is enhanced to 10 colors (7). Here we investigated, whether 10 colors are more predictive for the lineage dominance in GCs than single allele Confetti mice. It turns out that the correlation of lineage dominance and the product of color dominance and stained fraction of cells is rather similar with 4 or 10 colors (compare Figure 8 to Figures 5A,B and Figure 7). In particular, in one-shot stainings it is found that the predictive power of 4 colors is even better than with 10 colors for early stainings and low fractions of stained cells (Figures 8A,C). Further, for simulations with decaying tamoxifen-activity, the optimal time point (day 3 post GC onset) for the initiation of staining is the same as for 10 colors and the overall correlation is reduced to a similar degree (Figure 8B). This can be rescued by reducing the fraction of intially stained cells (Figure 8D).

The unexpected result that 10 colors would not be substantially better predictors than 4 colors prompted us to test whether the reason for this lack of improvement by more colors would be related to the rather inhomogeneous probability of activation of the different colors (Table 1). Indeed, when assuming equal probabilities for all 10 colors, the predictive power is better than for 4 colors and also better than for 10 colors based on real activation probabilities in Table 1 (compare Figure 8B, dashed and full lines).

## 4. Discussion

The present analysis supports that the Brainbow construct is suitable for the analysis of an evolutionary system like the evolution of GC B cells in the context of an immune response. When all germline B cells are stained before the GC reaction, the color distribution during and at the end of the evolutionary process in the GC reaction is predictive of the clonal distribution. Thus, staining and fate monitoring of colors is a good approximation for the analysis of clonal evolution. Sequencing of B cells is only necessary to account for specific clones, but the clonal dominance is well evaluated based on the colors alone.

When the Cre-dependent recombination is employed to stain the B cells in the course of a GC reaction, the color is predictive of the clonal composition provided the staining is limited to a rather short time interval. The longer the staining progresses, the weaker the correlation to the clonal distribution. With a prolonged staining procedure, staining ambiguities like staining of two lineages with the same color, is aggravated by the variability of initial states of the color distributions. A lineage is defined at the beginning of the staining procedure, such that a late staining event leads to a few stained cells in comparison to an early staining event, where the whole lineage branch would be stained. For that reason, it is recommended to limit the action of tamoxifen to a short time period.

An important source of ambiguity is that there is only a fraction of cells stained by tamoxifen-induced recombination. This implies that there is a proportion of GCs in which the unstained B cells would become dominant. A good predictive power relies on the elimination of black-dominated GCs, which can be achieved by imposing a staining threshold. This threshold eliminates all poorly stained GCs from the analysis. A suitable staining threshold is in the range of 40%. With longer staining periods, 50% leads to better results. A staining threshold of 45% was identified that allows to extrapolate the lineage dominance derived from the subset of GCs above the staining threshold to the whole set of measured GCs, including the GCs left out from the analysis.

Intuitively, one would expect that staining more B cells would improve the predictive power. This is only true without a staining threshold, thus, including all GCs irrespective how strongly they are stained. However, together with imposing a staining threshold, the relationship is inverted. Low staining fractions are substantially more predictive of clonal dominance than high staining fractions. At the same time, the total number of GCs eliminated by the threshold increases if only a small fraction of B cells per GC is stained. Thus, in the future design of such experiments it is important to find the right balance between statistical significance and low staining. Staining of 30% of the GC B cells appears as a good starting point from the point of view of *in silico* GCs.

The time point of staining is an important parameter. The general tendency is the earlier the better. At later time points, a pre-selected set of B cells is stained, which increases errors of staining different lineages with the same color, thus, further over-estimating the clonal dominance by the color dominance. This happens because the pre-selected B cells are more likely to both survive and persist in the continuation of the GC reaction. However, at very early time points, the set of different lineages is limited. Physiologically, it might make sense to wait until a minimum of B cell diversity was achieved. Otherwise, it would make more sense to use the Mx1-Cre system, in which founder GC B cells carry a color already when they start the GC reaction.

Depending on the question under consideration, one might want to analyse the set of cells present in the GC at the particular time point at which staining is induced, or of all cells getting stained in the course of the GC reaction. In the latter case, earlier staining increases predictive power. In the former case, earlier staining reduces predictive power. This is due to late founder cells that enter the *in silico* GC reaction and still get stained. Colored B cells exist without any lineage counterpart in the analysis. If lineages defined at a particular time point are in the focus of the research, there exists a time point of inducing the staining between day 2 and 3 post GC onset, which is optimal for the analysis of lineage dominance.

The analysis is based on a set of 1,000 *in silico* GC simulations in agreement with data from two-photon imaging (22–24) and anti-DEC205-OVA experiments (15). This set of GC simulations was used to interpret the data in Tas et al. (7) and correlates well with the results therein. Clonal and color dominance are likely depending on model parameters like affinity of founder cells to the antigen, division rate, selection pressure, strength of antibody feedback, etc. A different set of simulations might well quantitatively shift one or the other result, provided the change would influence the timing of selection or the number of mutations in the GC reaction. For example, the quality of the GC founder cells might vary depending on the type of immunization and the existence of transgenic B cells with a particular affinity to the immunizing antigen. This implies that the planning of new experiments would ideally go hand-in-hand with a specifically adapted set of simulations.

## Author contributions

MM-H and GV initiated the study. MM-H designed the C++ code and performed the analysis. SB contributed to R-coding and to the strategy of the data analysis. MM-H wrote the manuscript. All authors contributed to the scientific content and interpretation and revised the manuscript.

### Conflict of interest statement

The authors declare that the research was conducted in the absence of any commercial or financial relationships that could be construed as a potential conflict of interest.

## Code for the analysis

https://figshare.com/articles/Computer_Simulation_of_MultiColor_Brainbow_Staining_and_Clonal_Evolution_of_B_Cells_in_Germinal_Centers/7068419
