# Supplementary material for: Computer Simulation of Multi-Color Brainbow Staining and Clonal Evolution of B Cells in Germinal Centers
Source: Front Immunol. 2018 Sep 25;9:2020. doi: 10.3389/fimmu.2018.02020 (PMC6167470; doi:10.3389/fimmu.2018.02020)
Supplement: Supplementary file 1 [file Data_Sheet_1.pdf]

frontiers

Supplementary Material: Computer simulation of multi-colour Brainbow staining and clonal evolution of B cells in germinal centres

1 GC MODEL ASSUMPTIONS

The GC model assumptions underlying the germinal centre (GC) simulations are explained here and include the used parameter values. It follows the description of (Meyer-Hermann et al., 2012) adapted to include novel features introduced since then in (Meyer-Hermann, 2014; Binder and Meyer-Hermann, 2016). Used acronyms are: DZ for dark zone, LZ for light zone, Tfh for T follicular helper cell, FDC for follicular dendritic cell.

Space representation

All reactions take place on a three-dimensional discretized space with a rectangular lattice with lattice constant of $\Delta x = 5\mu m$. Every lattice node can be occupied by a single cell only.

Shape space for antibodies

Antibodies are represented on a four dimensional shape space (Perelson and Oster, 1979). The shape space is restricted to a size of 10 nodes per dimension, thus, only considering antibodies with a minimum affinity to the antigen. The optimal clone Φ^* is positioned in the centre of the shape space. A position on the shape space Φ is attributed to each B cell. Its Hamming distance $||\Phi - \Phi^*||_1$ to the optimal clone is used as a measure for the antigen binding probability. The binding probability is calculated from the Gaussian distribution with width $\Gamma = 2.8$ (Meyer-Hermann et al., 2001):

$$b(\Phi, \Phi^*) = \exp\left(-\frac{||\Phi - \Phi^*||_1^2}{\Gamma^2}\right) . \quad \text{(S1)}$$

B cell phenotypes

Three B cell phenotypes are distinguished: DZ B cells, LZ B cells, and output cells. The different phenotypes characterize the cell properties and are not meant as localization within the GC zones. DZ B cells divide, mutate and migrate. LZ B cells also migrate and undergo the different stages of the selection process. Output cells only migrate.

Founder cells

The model starts from 250 Tfh, 200 FDCs, 300 stromal cells, and no B cell. Tfh are randomly distributed on the lattice and occupy a single node each. Stromal cells are restricted to the DZ (see section *Chemokine distribution* for their function). FDCs are restricted to the upper half of the reaction sphere, occupy one node by their soma and have 6 dendrites of $40\mu m$ length each. The presence of dendrites is represented as a lattice-node property and, thus, visible to B cells. The dendrites are treated as transparent for B

cell or Tfh migration such that they do not inhibit cell motility. Each FDC is loaded with 3000 antigen portions distributed onto the lattice-nodes occupied by FDC-soma or FDC-dendrite. One antigen portion corresponds to the number of antigen molecules taken up by a B cell upon successful contact with an FDC.

B cell influx rate

As B cell selection is not active during the first 3 days of the reaction (i.e. days 3-5 post immunisation), the first 3 days can be approximated as a B cell expansion phase. Clonality can be safely ignored, and it suffices to consider a single dividing cell type B_i, where i denotes the generation of the B cells. The dynamics of expansion are then described by

$$\begin{aligned} \frac{dB_1}{dt} &= s - pB_1 \\ \frac{dB_\mathrm{i}}{dt} &= 2pB_{\mathrm{i}-1} - pB_\mathrm{i} \quad \text{for } i > 1 \\ \frac{dB_\mathrm{GC}}{dt} &= 2pB_{\mathrm{i}_\mathrm{max}}\,, \end{aligned} \tag{S2}$$

where s is the influx rate, p the division rate, i_max the number of divisions per cell in the expansion phase, and B_GC the resulting number of GC B cells that participate in the GC reaction. The number of initial divisions is estimated by the maximum number of divisions observed upon anti-DEC205-OVA treatment, i.e. $i_\mathrm{max} = 6$ (Victora et al., 2010; Meyer-Hermann et al., 2012). As the division time $\ln(2)/p$ is shorter than the expansion phase $T_\mathrm{expand} = 3$ days, one may solve Eq. (S2) in steady state, yielding:

$$B_6 = 2B_5 = 4B_4 = 8B_3 = 16B_2 = 32B_1 = 32s/p\,. \tag{S3}$$

Thus, the relevant ODE becomes

$$\frac{dB_\mathrm{GC}}{dt} = 2pB_6 = 64s \implies B_\mathrm{GC} = 64st\,, \tag{S4}$$

i.e. a linear growth in time proportional to the constant influx during expansion. Note that in the steady state approximation the influx rate becomes independent of the division rate p, which is an implication of the assumption of a fixed number of divisions per founder cell i_max. With the side condition of getting 9000 cells at day 3, $B_\mathrm{GC}(T_\mathrm{expand} = 72\mathrm{hr}) = 9000$, the influx rate is estimated to be

$$s = \frac{B_\mathrm{GC}(T_\mathrm{expand})}{64T_\mathrm{expand}} \approx 2\ \mathrm{cells/hr}\,. \tag{S5}$$

This corresponds to 144 B cells entering the GC in the first 3 days of expansion and building up the founder cell population of the GC reaction.

Motivated by this estimation, in the model we assumed that B cells enter the GC reaction with a probability corresponding to a rate of 2 cells per hour. New B cells are randomly positioned on the lattice (exclusively on free nodes). The shape space position of each new B cell is randomly picked from a set of 100 shape space positions, which are randomly chosen on the shape space.

DZ B cell division

The average cell cycle duration of 7 hours of DZ B cells is varied for each B cell according to a Gaussian distribution. This is needed to get desynchronization of B cell division. The cell cycle is decomposed into four phases (G1, S, G2, M) in order to localize mitotic events if this is needed.

Each founder B cell divides a number of times before differentiating to the LZ phenotype for the first time. Six divisions was the number of divisions found in response to the extreme stimulus with anti-DEC205-OVA (Victora et al., 2010; Meyer-Hermann et al., 2012). Each selected B cell divides an number of times determined by the interaction with Tfh (see below, LZ B cell selection). The parameters of the interaction with Tfh are tuned such that the mean number of divisions is in the range of two (Gitlin et al., 2014). This value is required in order to maintain a DZ to LZ ratio in the range of two (Victora et al., 2010; Meyer-Hermann et al., 2012).

A division requires free space on one of the Moore neighbors of the dividing cell. Otherwise the division is postponed until a free Moore neighbor is available.

At every division the encoded antibody can mutate with a probability of 0.5 (Berek and Milstein, 1987; Nossal, 1992). This corresponds to a shift in the shape space to a von Neumann neighbor in a random direction. Upon selection by Tfh the mutation probability is individually reduced from $m_{\max} = 0.5$ down to $m_{\min} = 0$ in an affinity-dependent way following

$$m(b) \quad = \quad m_{\max} - (m_{\max} - m_{\min})\, b \tag{S6}$$

with b from Eq. (S1) (Toellner et al., 2002). Thus, after recycling DZ B cells can acquire reduced mutation probabilities. This mechanism is motivated by the observation that B cell receptor internalization enhances the activation of the kinase Akt (Chaturvedi et al., 2011) which, in turn, suppresses activation induced cytosine deaminase (AID) (Omori et al., 2006). AID is required for somatic hypermutation, such that this provides an affinity-dependent down-regulation of the mutation frequency (Dustin and Meyer-Hermann, 2012). However, there is no formal proof of this mechanism.

B cell division of B cells that previously acquired antigen and have been selected by Tfh distribute the retained antigen asymmetrically to the daughters (Thaunat et al., 2012). The model assumes asymmetric division in 72% of the cases, which is supported by experimental observations (see Thaunat et al. (2012) and Supplementary Figure S1 in Meyer-Hermann et al. (2012)). If division is asymmetric, one daughter gets all the retained antigen while the other gets none, which approximates the value of 88% found in (Thaunat et al., 2012). Mutation is suppressed in asymmetric divisions, which is an arbitrary choice.

After the required number of divisions the B cell differentiates with a rate of in $1/6$ minutes to the LZ phenotype. All B cells that kept the antigen up to this time, differentiate to output cells, up-regulate CXCR4, and leave the GC in direction of the T zone. The alternatives, that B cells randomly differentiate to output cells after divisions with a probability of 23% (LEDAX model in Meyer-Hermann et al. (2012)) or that B cells decide to differentiate to output cells right after interaction with Tfh (BASE mode in Meyer-Hermann et al. (2012)), leads to very similar GC readouts. However, the amount of generated output cells is substantially higher if the B cells differentiate to output cells after divisions as compared to after selection (Dustin and Meyer-Hermann, 2012).

LZ B cell selection

LZ B cells can be in the states *unselected, FDC-contact, FDC-selected, Tfh-contact, selected, apoptotic*.

Unselected

LZ B cells migrate and search for contact with FDCs loaded with antigen in order to collect antigen for 0.7 hours. If an FDC soma or dendrite is present at the position of the B cell, the B cell attempts to establish contact to the FDC with probability b in Eq. (S1). If the available number of antigen portions at the specific FDC site drops below 20 the binding probability b is linearly reduced with the number of available portions. If successful, the B cell switches to the state *FDC-contact*; otherwise the B cell continues to migrate. Further binding-attempts are prohibited for 1.2 minutes. At the end of the antigen collection period, B cells switch to the state *FDC-selected*. If a LZ B cell fails to collect any antigen at this time it switches to the state *apoptotic*.

FDC-contact

LZ B cells remain immobile (bound) for 3 minutes (Schwickert et al., 2007) and then return to the state *unselected*. The counter for the number of successful antigen uptake events is increased by one and the FDC reduces its locally available antigen portions by one.

FDC-selected

B cells search for contact with Tfh. If they meet a Tfh they switch to the state *TC-contact*.

Tfh-contact

LZ B cells remain immobile for 36 minutes. In this time the bound Tfh, which may also be bound to other B cells, polarizes to the bound B cell with highest number of successful antigen uptakes. After the binding time, the B cell switches to the state *apoptotic* if the Tfh was polarized for less than 30 minutes to it. Otherwise it switches to the state *selected*. Note that other variants of the model exist in which a B cell interacts with multiple Tfh for shorter times (Wang et al., 2016; Papa et al., 2017) and collect signals from these interactions before fate decision.

Selected

LZ B cells keep the LZ phenotype for six hours and desensitize for CXCL13, thus, perform a random walk. During that time they re-enter cell cycle and progress through the cell cycle phases. Then they recycle back to the DZ phenotype with a rate of $1/6$ minutes and memorize the amount of collected antigen as well as the cell cycle phase they have achieved by this time.

The number of divisions $P(A)$ the recycled B cells will do is derived from the amount of collected antigen A, which reflects the amount of pMHC presented to Tfh and the affinity of the B cell receptor for the antigen, as follows:

$$P(A) \quad = \quad P_{\min} + (P_{\max} - P_{\min}) \, \frac{A^{n_\mathrm{P}}}{A^{n_\mathrm{P}} + K_\mathrm{P}^{n_\mathrm{P}}} \; . \tag{S7}$$

The more antigen was collected by the B cell, the more divisions are induced. We set the minimum number of division to one ($P_{\min} = 1$) in order to avoid recycling events without further division. It is limited by six divisions in the best case, which is motivated by anti-DEC205-OVA experiments in which DEC205$^{+/+}$ B cells received abundant antigen which increased pMHC presentation to a maximum Victora et al. (2010). The population dynamics in vivo and in silico only matched when the number of divisions was increased to six in the simulation Meyer-Hermann et al. (2012) suggesting that the strongest

possible pMHC presentation to Tfh induces six divisions ($P_{\max} = 6$). The Hill-coefficient was set to $n_{\rm P} = 2$.

The half value $K_{\rm P}$ remained to be determined, which denotes the amount of antigen collected by B cells at which the number of divisions becomes half maximal. The number of collected antigen portions varies between zero and a maximum determined by the duration of the antigen collection phase, the duration of each B cell interaction with FDCs, and the migration time between two antigen presenting sites. The numbers of successful B cell-FDC encounters as observed in the simulations served as estimate of $A_{\max}$. Low affinity B cells had zero or one antigen uptake event, while high affinity cells took up between 5 and 10 portions. For an intermediate antigen uptake of $A_0 = 4.5$, the resulting number of divisions has to be $P_0 = 2$ in order to be in agreement with the mean number of divisions in the range of two (Gitlin et al., 2014), which leads to the condition:

$$K_{\rm P} \;\approx\; A_0 \left(\frac{P_{\max} - P_{\min}}{P_0 - P_{\min}} - 1 \right)^{1/n_{\rm P}} \;=\; 9 \quad . \tag{S8}$$

Apoptotic

LZ B cells remain on the lattice for 6 hours before they are deleted. They continue to be sensitive to CXCL13 during this time.

Chemokine distribution

Two chemokines CXCL12 and CXCL13 are considered. CXCL13 is produced by FDCs in the LZ with 10nMol per hour and FDC while CXCL12 is produced by stromal cells in the DZ with 400nMol per hour and stromal cell. As both cell types are assumed to be immobile, chemokine distributions were pre-calculated once and the resulting steady state distributions were used in all simulations.

Chemotaxis

DZ and LZ B cells regulate their sensitivity to CXCL13 and CXCL12, respectively. This is true in all B cell states unless stated otherwise. All B cells move with a target speed of $7.5\mu m/min$. This leads to a slightly lower observable average speed of $\approx 6\mu m/min$.

B cells have a polarity vector that determines their preferential direction of migration. The polarity vector $\vec{p}$ is reset every 1.5 minutes into a new direction using the chemokine distribution c as

$$\vec{p} \;=\; \vec{p}_{\rm rand} + \frac{\alpha}{1 + \exp\left\{ \kappa \left(K_{1/2} - \Delta x |\vec{\nabla} c| \right) \right\}} \frac{\vec{\nabla} c}{|\vec{\nabla} c|} \quad , \tag{S9}$$

where $\vec{p}_{\rm rand}$ is a random polarity vector and the turning angle is sampled from the measured turning angle distribution ((Allen et al., 2007) Fig. S1B). $\alpha = 10$ determines the relative weight of the chemotaxis and random walk, $K_{1/2} = 2 \cdot 10^{11}$ Mol determines the gradient of half maximum chemotaxis weight, and $\kappa = 10^{10}/{\rm Mol}$ determines the steepness of the weight increase.

B cells de- and re-sensitize for their respective chemokine depending on the local chemokine concentration: The desensitization threshold is set to 6nMol and 0.08nMol for CXCL12 and CXCL13,

respectively, which avoids cell clustering in the center of the zones. The resensitization threshold is set at $2/3$ and $3/4$ of the desensitization threshold for CXCL12 and CXCL13, respectively.

B cells can only migrate if the target node is free. If occupied and the neighbor cell is to migrate in the opposite direction (negative scalar product of the polarity vectors) both cells are exchanged with a probability of 0.5. This exchange algorithm avoids lattice artifacts leading to cell clusters.

Tfh do random walk with a preferential directionality to the LZ: The polarity vector $\vec{p}$ of Tfh is determined from a mixture of random walk $\vec{r}$ and the direction of the LZ $\vec{n}$ by

$$\vec{p} \;\; = \;\; (1-\alpha')\vec{r} + \alpha'\vec{n}\;, \tag{S10}$$

where $\alpha' = 0.1$ is the weight of chemotaxis. This weight leads to a dominance of random walk with a tendency to accumulate in the LZ as found in experiment. TCs migrate with an average speed of $10\mu m/min$ and repolarize every 1.7 minutes (Miller et al., 2002).

Output cell motility is derived from plasma cell motility data to be $3\mu m/min$ (Allen et al., 2007) with a persistence time of 0.75 minutes.

REFERENCES

Allen, C. D., Okada, T., Tang, H. L., and Cyster, J. G. (2007). Imaging of germinal center selection events during affinity maturation. *Science* 315, 528–531

Berek, C. and Milstein, C. (1987). Mutation drift and repertoire shift in the maturation of the immune response. *Immunol. Rev.* 96, 23–41

Binder, S. and Meyer-Hermann, M. (2016). Implications of intravital imaging of murine germinal centers on the control of B cell selection and division. *Front. Immunol.* 7, 593

Chaturvedi, A., Martz, R., Dorward, D., Waisberg, M., and Pierce, S. K. (2011). Endocytosed BCRs sequentially regulate MAPK and Akt signaling pathways from intracellular compartments. *Nat. Immunol.* 12, 1119–1126

Dustin, M. and Meyer-Hermann, M. (2012). Antigen feast or famine. *Science* 335, 408–409

Gitlin, A. D., Shulman, Z., and Nussenzweig, M. C. (2014). Clonal selection in the germinal centre by regulated proliferation and hypermutation. *Nature* 509, 637–640

Meyer-Hermann, M. (2014). Overcoming the dichotomy of quantity and quality in antibody responses. *J. Immunol.* 193, 5414–5419

Meyer-Hermann, M., Deutsch, A., and Or-Guil, M. (2001). Recycling Probability and Dynamical Properties of Germinal Center Reactions. *J. Theor. Biol.* 210, 265–285

Meyer-Hermann, M., Mohr, E., Pelletier, N., Zhang, Y., Victora, G. D., and Toellner, K.-M. (2012). A theory of germinal center B cell selection, division, and exit. *Cell Rep.* 2, 162–174

Miller, M., Wei, S., Parker, I., and Cahalan, M. (2002). Two-photon imaging of lymphocyte motility and antigen response in intact lymph node. *Science* 296, 1869–73

Nossal, G. (1992). The molecular and cellular basis of affinity maturation in the antibody response. *Cell* 68, 1–2

Omori, S. A., Cato, M. H., Anzelon-Mills, A., Puri, K. D., Shapiro-Shelef, M., Calame, K., et al. (2006). Regulation of Class-Switch Recombination and Plasma Cell Differentiation by Phosphatidylinositol 3-Kinase Signaling. *Immunity* 25, 545–557

Papa, I., Saliba, D., Ponzoni, M., and Bustamante, S. e. a. (2017). Tfh-derived dopamine accelerates productive synapses in germinal centres. *Nature* 547, 318–323

Perelson, A. S. and Oster, G. F. (1979). Theoretical Studies of Clonal Selection: Minimal Antibody Repertoire Size and Reliability of Self-Non-self Discrimination. *J. Theor. Biol.* 81, 645–670

Schwickert, T. A., Lindquist, R. L., Schakhar, G., Livshits, G., Skokos, D., Kosco-Vilbois, M. H., et al. (2007). In vivo imaging of germinal centres reveals a dynamic open structure. *Nature* 446, 83–87

Thaunat, O., Granja, A. G., Barral, P., Filby, A., Montaner, B., Collinson, L., et al. (2012). The asymmetric segregation of polarized antigen on B cell division shapes presentation capacity. *Science* 335, 475–479

Toellner, K. M., Jenkinson, W. E., Taylor, D. R., Khan, M., Sze, D. M. Y., Sansom, D. M., et al. (2002). Low-level Hypermutation in T Cell-independent Germinal Centers Compared with High Mutation Rates Associated with T Cell-dependent Germinal Centers. *J. Exp. Med.* 195, 383–389

Victora, G. D., Schwickert, T. A., Fooksman, D. R., Kamphorst, A. O., Meyer-Hermann, M., Dustin, M. L., et al. (2010). Germinal center dynamics revealed by multiphoton microscopy with a photoactivatable fluorescent reporter. *Cell* 143, 592–605

Wang, P., Chang-ming, S., Qi, H., and Lan, Y.-h. (2016). A stochastic model of the germinal center integrating local antigen competition, individualistic T-B interactions, and B cell receptor signaling. *J Immunol* 197, 1169–1182

2 SUPPLEMENTARY FIGURES

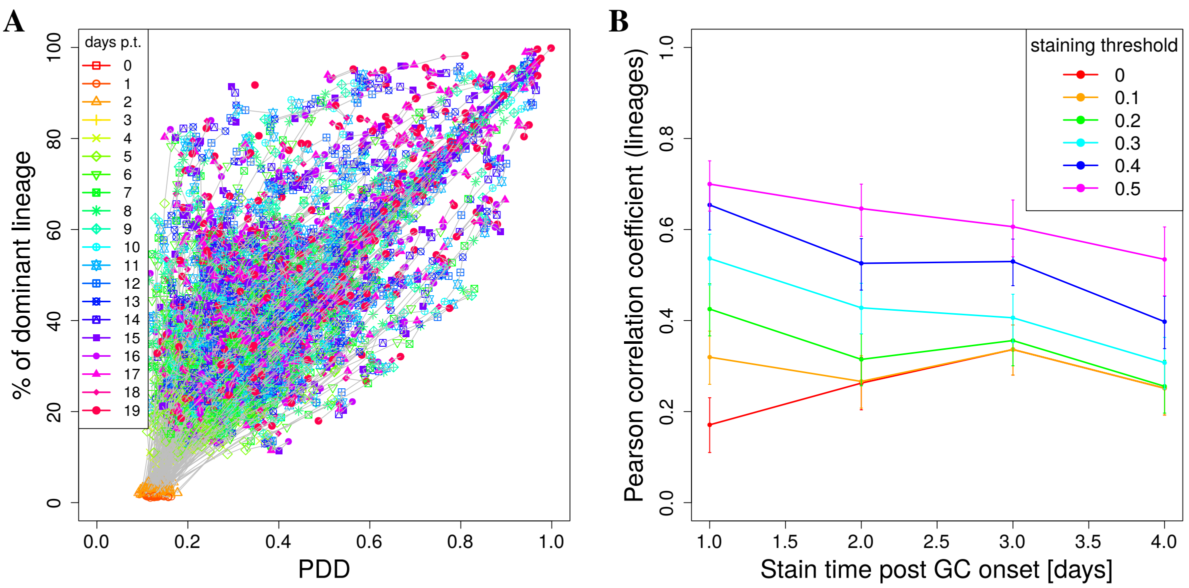

Figure S1: **Including late founder cells in the set of lineages.** Correlation between lineage and the product of colour dominance and colour density (PDD) in simulations with tamoxifen decay. Lineages are defined by all cells exisiting at the time of staining initiation and are complemented by all founder cells entering the GC reaction after this time. Tamoxifen-induced staining with 10 colours (Tab. 1) and decay of tamoxifen-induced staining with half life of $\tau_{\text{tamoxifen}} = 24$ hours in Eq. (3) was assumed. **(A):** Staining was started at day 1 post GC onset with staining threshold of 40%. Colours depict the day post tamoxifen (p.t.). **(B):** The starting time and the staining threshold were varied. Pearson correlation coefficient from 1000 *in silico* GCs at day 11 post tamoxifen. 95% approximate confidence intervals to the Pearson product moment correlation were computed using the Fisher transformation.

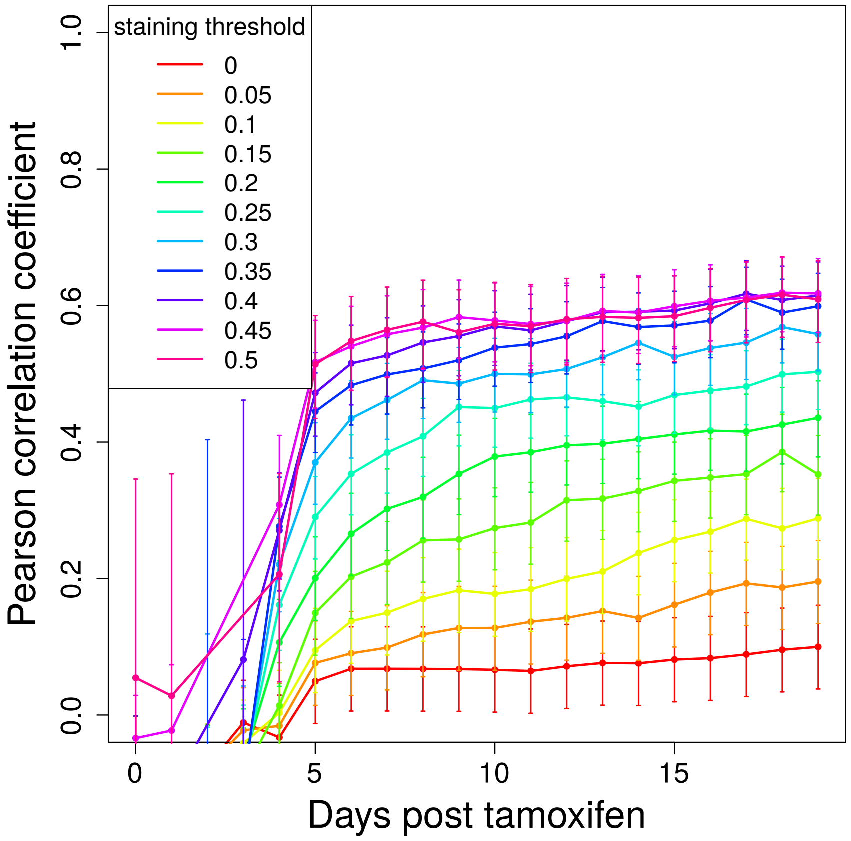

Figure S2: **Correlation of lineage dominance and colour density.** Correlation between lineage dominance and colour density in simulations with one-shot induction of colour expression by tamoxifen at day 2 post GC onset with different imposed staining thresholds. Lineages are defined by all GC B cells exisiting at this time. Tamoxifen-induced staining with 10 colours (Tab. 1). Pearson correlation coefficient from 1000 *in silico* GCs. 95% approximate confidence intervals to the Pearson product moment correlation were computed using the Fisher transformation.

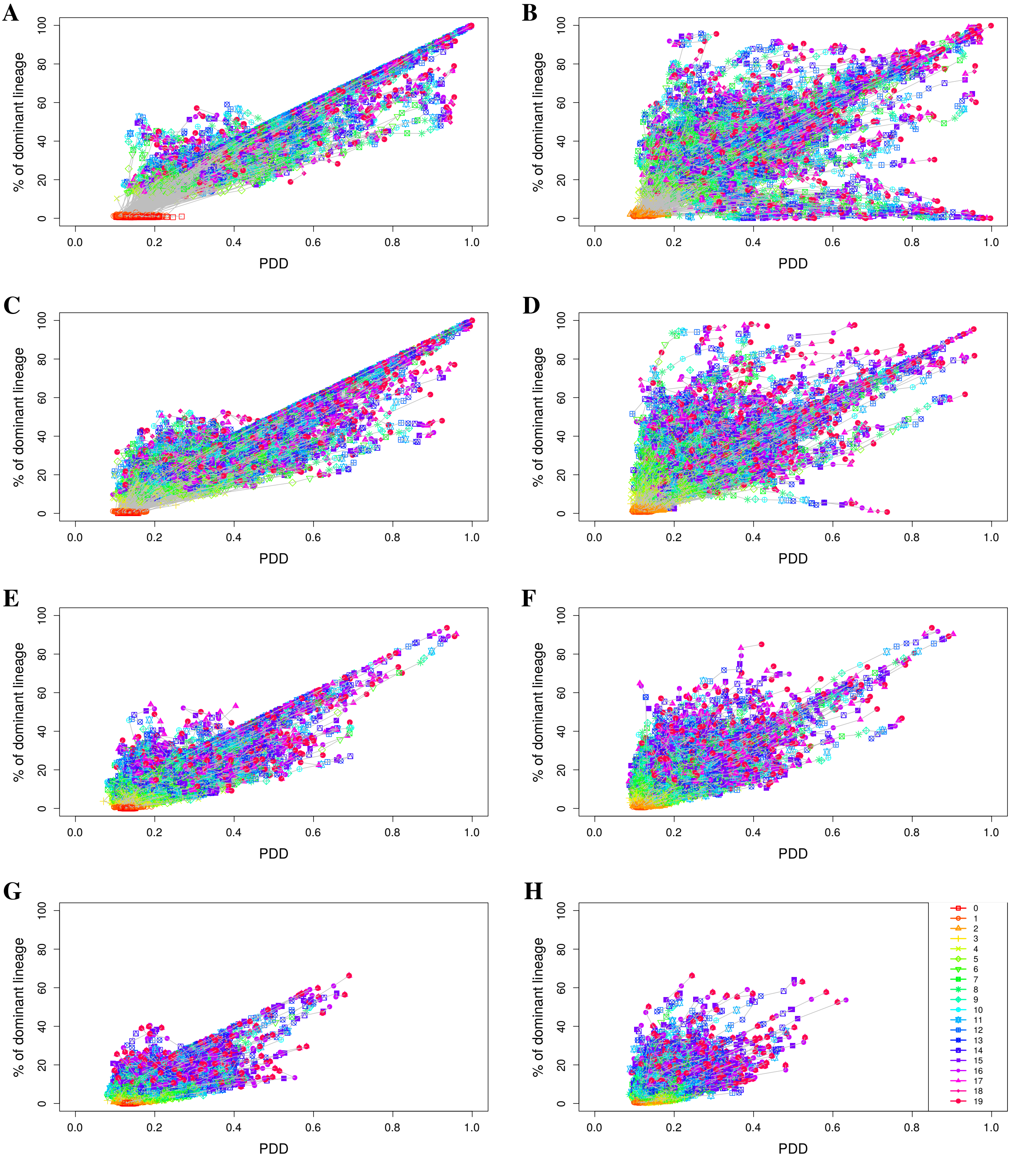

Figure S3: **With tamoxifen decay colour under-estimates lineage dominance.** Correlation between lineage and the product of colour dominance and colour density (PDD) in simulations with single shot **(left panels)** or dynamic decay **(right panels)** of tamoxifen-induced staining with 10 colours (Tab. 1). For dynamic tamoxifen decay a half life of $\tau_{\text{tamoxifen}} = 24$ hours was assumed in Eq. (3). Lineages were defined as the set of all cells existing at the start of staining. Staining was started at day 1 **(A)**,**(B)**, 2 **(C)**,**(D)**, 3 **(E)**,**(F)**, and 4 **(G)**,**(H)** post GC onset. A staining threshold of 40% was assumed in all panels. The time point in the GC reaction was distinguished (symbol colours). Pearson correlation coefficient and 95% confidence interval from 1000 *in silico* GCs.

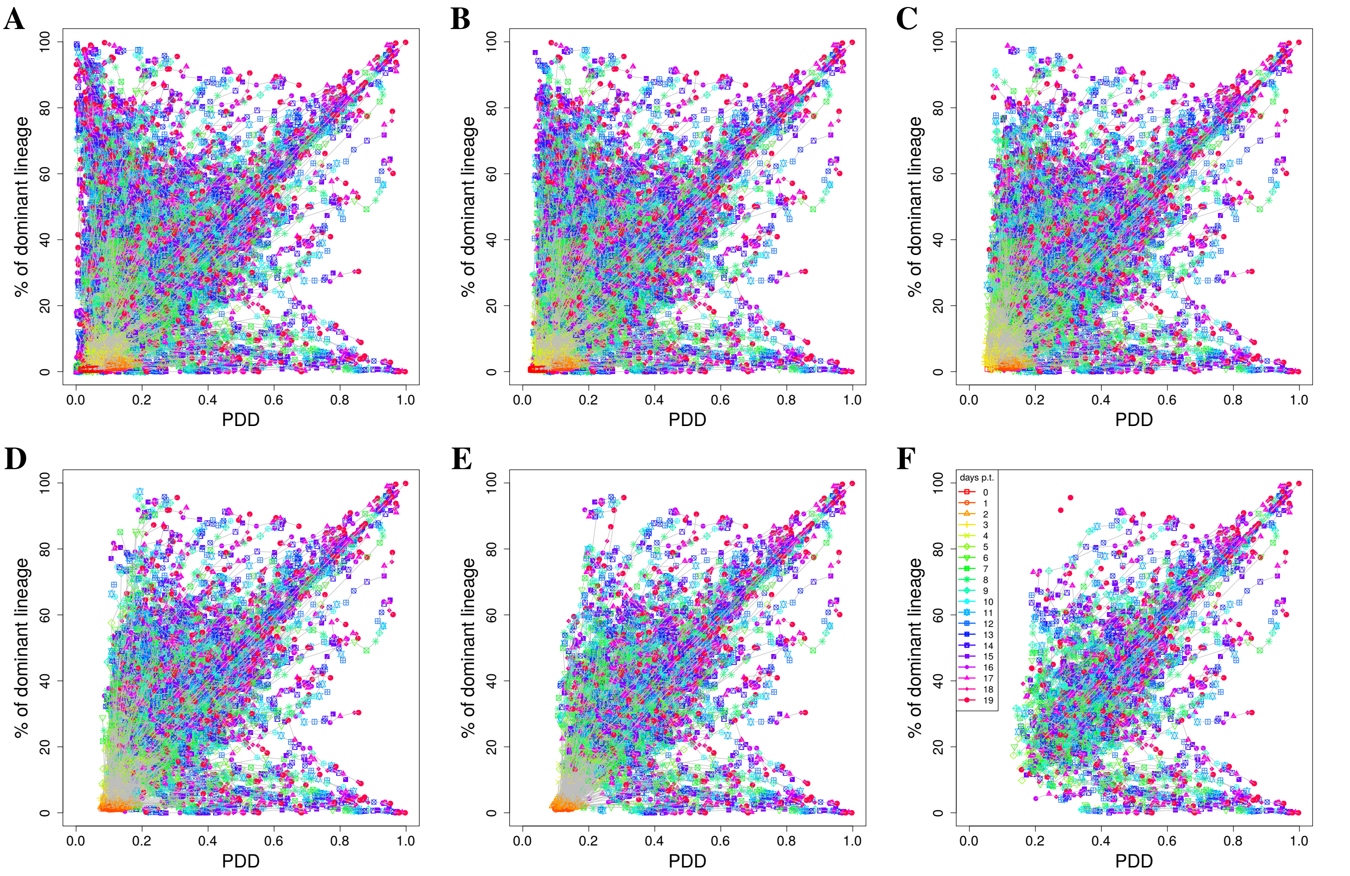

Figure S4: **With tamoxifen decay the staining threshold fails to remove colour under-estimation.** Correlation between lineage and the product of colour dominance and colour density (PDD) in simulations with decay of tamoxifen-induced staining with 10 colours (Tab. 1) in 1000 *in silico* GCs. Staining was started at day 1 post GC onset with staining threshold of **(A)** 0, **(B)** 10, **(C)** 20, **(D)** 30, **(E)** 40, and **(F)** 50%. A tamoxifen half life of $\tau_{\text{tamoxifen}} = 24$ hours was assumed in Eq. (3). Lineages were defined as the set of all cells existing at the start of staining. The time point in the GC reaction was distinguished (symbol colours, days post tamoxifen (p.t.)).

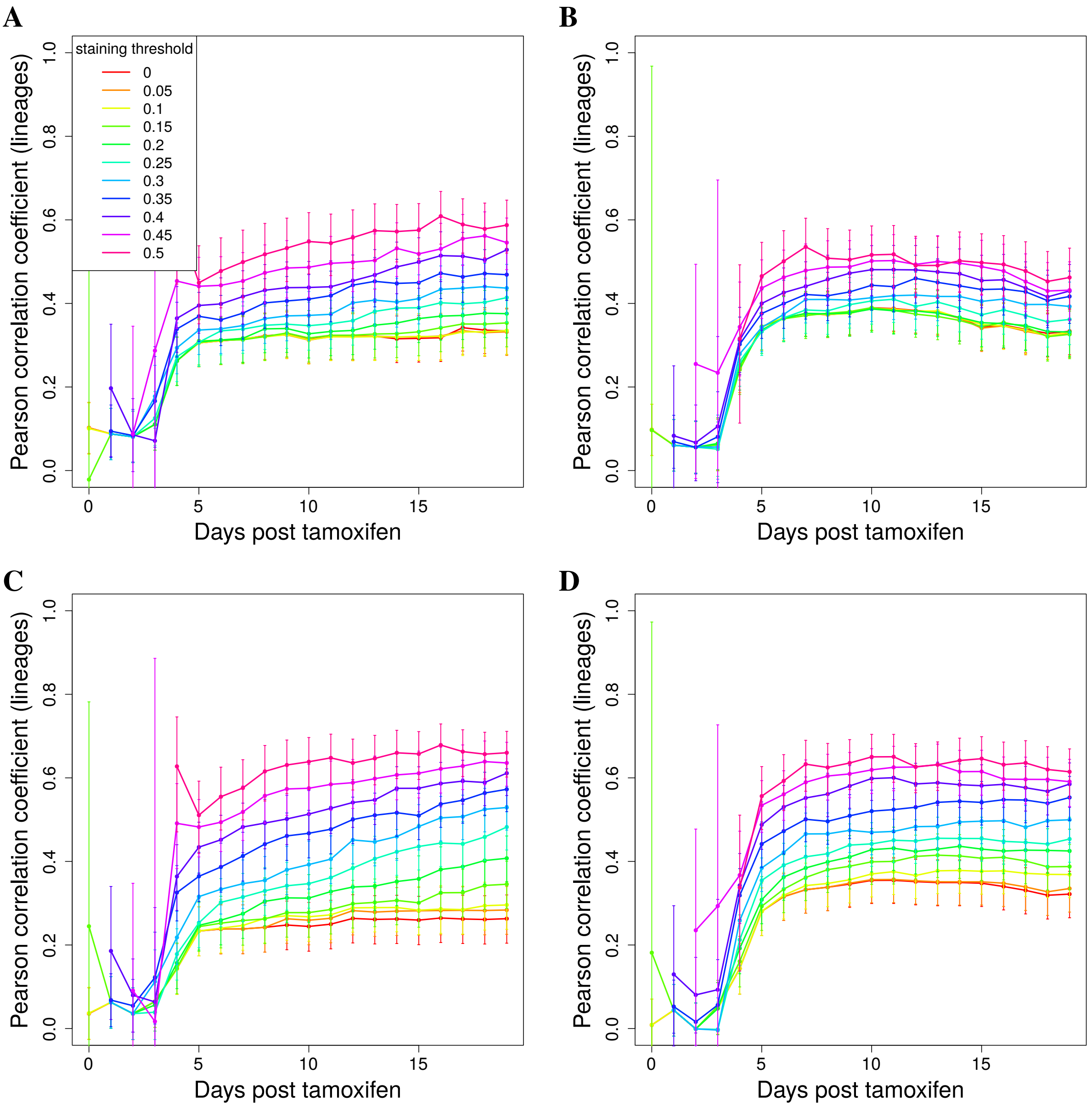

Figure S5: **Impact of recombination of already recombined cells.** Correlation between lineage and colour dominance (A,B) or PDD (C,D) in simulations with decay of tamoxifen-induced staining with 10 colours (Tab. 1) and with a tamoxifen half life of $\tau_{\text{tamoxifen}} = 24$ hours in Eq. (3). Simulations without (A,C) and with (B,D) the possibility of recombination of cells, which already recombined and expressed a colour during the period of action of tamoxifen. Staining was started at day 2 post GC onset and lineages were defined as the set of all cells existing at the start of staining. Pearson correlation coefficient from 1000 *in silico* GCs with different staining thresholds (line colours). 95% approximate confidence intervals to the Pearson product moment correlation were computed using the Fisher transformation.

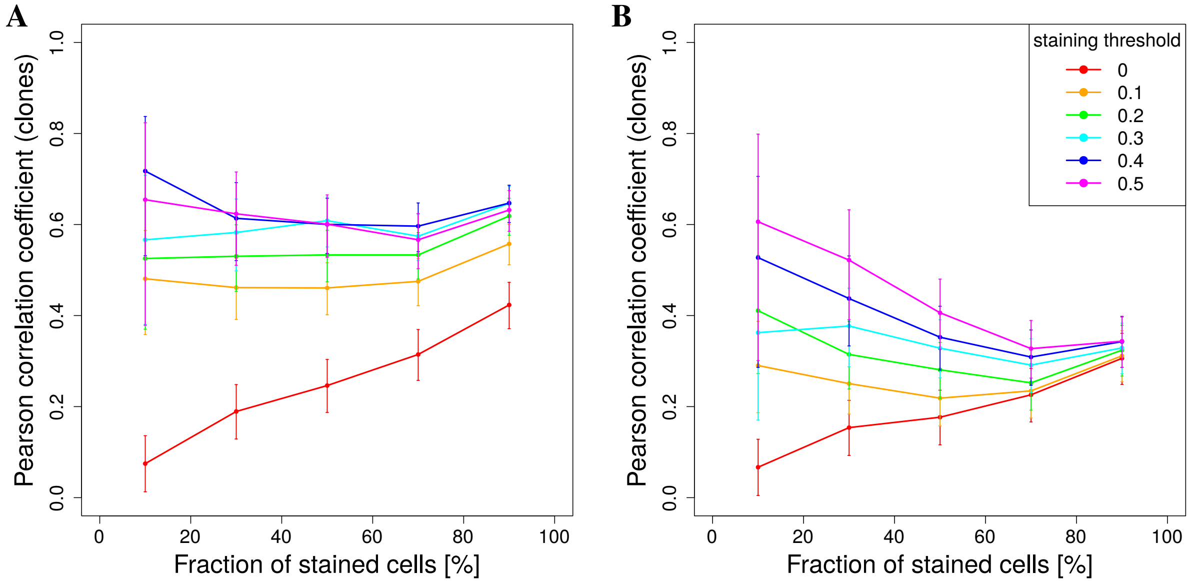

Figure S6: **Tamoxifen-induced staining has limited predictive power of clonal dominance.** Correlation between clonal dominance and the product of colour dominance and colour density in dependence on the fraction of stained cells. Simulations with single shot staining with 10 colours (Tab. 1) at day 1 **(A)** or day 2 **(B)** post GC onset. Pearson correlation coefficient from 1000 *in silico* GCs at day 11 post tamoxifen with different staining thresholds (line colours). 95% approximate confidence intervals to the Pearson product moment correlation were computed using the Fisher transformation.
